# Enhancing care transition performance of community pharmacies in Nigeria

**DOI:** 10.1002/hsr2.1904

**Published:** 2024-02-15

**Authors:** Adeboye O. Bamgboye, Ibrahim A. Hassan, Elijah O. Fatoye, Chioma C. Ozuluoha, Samson O. Folami, Theogene Uwizeyimana

**Affiliations:** ^1^ Faculty of Pharmacy Obafemi Awolowo University Ile‐Ife Nigeria; ^2^ Medical Centre, Lagos State Health Service Commission University of Lagos Lagos Nigeria; ^3^ Faculty of Pharmacy University of Uyo Uyo Nigeria; ^4^ Nigerian Institute of Medical Research Lagos Nigeria; ^5^ Bill & Joyce Cummings Institute of Global Health University of Global Health Equity Butaro Rwanda; ^6^ Department of Public Health Mount Kenya University Kigali Rwanda

**Keywords:** community pharmacy, intervention, Nigeria, primary care, referral, transition of care

## Abstract

Community pharmacies (CPs) represent a crucial source of primary care for the Nigerian population. Pharmacists in this setting provide essential primary care services to the public and, when required, facilitate care transitions or referrals for patients to higher levels of care. Given their accessibility and expanding roles in patient care, pharmacists are considered pivotal to meeting the transition of care (TOC) goals, that is, continuity, quality, and safety, especially at the community level. However, CPs in Nigeria face significant systemic and human‐factor barriers that impede their TOC performance, especially with their exclusion from the national care referral framework. Removing these barriers is essential to avoid the health consequences of a breakdown in the TOC system for the patients receiving care at the CPs. This paper discusses the barriers to effective TOC performance of the CPs in Nigeria and offers recommendations to address the deficiencies to improve patient care delivery using an inclusive and collaborative referral model.

## 1 INTRODUCTION

In Nigeria, community pharmacies (CPs) are a favorite point‐of‐call for health‐related concerns and an essential resource for primary healthcare (PHC) to the public.^1^ About a quarter to a half of the population seek care in the pharmacy.^2^ Community pharmacists in Nigeria offer extended care services across many PHC domains, including treating minor illnesses, medication counseling and management, health education, point‐of‐care tests, immunization, and family planning.^1^ Furthermore, community pharmacist‐led interventions have positively impacted disease prevention and drug‐related care outcomes across PHC settings.^3^, ^4^, ^5^ Given their expanding roles and direct access to many people at the grassroots level, community pharmacists are considered critical to achieving universal health coverage and other health‐related goals.^1^, ^6^ As expected, however, some of the health concerns of the patients at the pharmacy cannot be adequately addressed independently by the community pharmacists. A need for technical procedures, surgery, specialized psychiatric interventions, or specialist advice on diagnosis and treatment, among other interventions, often necessitates the involvement of other healthcare professionals in the care process for patients visiting the pharmacy.^7^ This creates the need for a referral or transition of care (TOC) system.

The TOC system describes the movement of patients from one healthcare provider or setting to another while ensuring the continuity, safety, and quality of care offered or received.^7^ However, care transitions are a potentially hazardous phase for some patients in the absence of guidance and cooperation of practitioners.^6^ It is estimated that, without support, discharged older patients may experience at least one medication‐related problem within the first 2 weeks in the community, potentially resulting in preventable readmission.^6^ Thus, according to the World Health Organization (WHO), ensuring optimal TOC processes and interventions at the primary care level is important, being the setting where most healthcare interventions are rendered.^8^ Globally, the role and impacts of community pharmacists in achieving the goals of TOC to‐and‐fro the primary care level is increasingly recognized. In a 2020 meta‐analysis of 39 studies conducted across Europe, North America, and Australia, community pharmacist interventions in hospital‐to‐home TOC were associated with over 40% reduction in the rates of hospital readmission within 30 days of discharge across different patient groups.^9^ Interestingly, the level of benefits recorded correlated with the extent of involvement and commitment of the pharmacists to the TOC process.^9^ Similarly, in another systematic review focusing on coronary artery disease care among US adults, Weeda et al. reported that patients who received pharmacist‐led TOC interventions such as medication reconciliation, counseling, and postdischarge monitoring had lower rates of emergency room visits or acute care needs than those who did not.^10^ Thus, further collaborative engagement of pharmacists in TOC is encouraged for better outcomes.

Ideally, the TOC process should be seamless, but the reality in Nigeria leaves much to be desired. A long list of barriers remain in the path to smooth TOC in Nigeria, including suboptimal service‐readiness of many PHC facilities,^11^ low transition‐preparedness of young patients for adult care,^12^ poor patient knowledge and perception about TOC,^13^ and prevalent patient self‐referral behavior.^14^ In the same vein, the care transition performance of CPs in Nigeria is challenged by factors relating to the non‐inclusive national referral framework, pharmacists' TOC practice, and patient attitudes to care transition. Nelissen et al. identified the lack of a formal collaborative working platform between community pharmacists and medical doctors as a major obstacle to maximizing the potential of pharmacy‐based hypertension care for patients.^15^ Unfortunately, such a breakdown in the TOC system can delay access to care, increase healthcare costs, stall diagnosis, and fuel therapy‐related problems such as drug interactions, inappropriate medication use, and consequent distrust in the health system.^7^ Therefore, there is a need for models that integrate CPs in Nigeria into the national care referral framework to enhance their TOC performance and patient care. This paper discusses barriers to the effective TOC performance of CPs in Nigeria and suggests mitigation strategies.

## 2 BARRIERS HINDERING EFFECTIVE REFERRAL/CARE TRANSITION PROCESS FROM THE CP IN NIGERIA

The barriers arise from systemic and human factors peculiar to Nigeria, requiring a context‐specific approach to surmount. The foremost barrier, in our view, is the nonrecognition‐cum‐exclusion of CPs as member facilities in the national healthcare referral framework in Nigeria.^16^ The Nigerian healthcare delivery system has three main levels: primary health centers, secondary facilities, and tertiary health institutions—featuring both public and private establishments. CPs are mostly private‐for‐profit health establishments but are influential in the PHC delivery value chain.^1^ The exclusion of CPs in Nigeria is deficient because the value of CPs in achieving effective, safe, timely, and patient‐centered PHC delivery is established and in accordance with current best practices worldwide.^1^, ^2^, ^3^, ^6^, ^7^ In our experience, however, providers at higher care levels seldom consider interventions from CPs in Nigeria. Community pharmacists are rarely privy to the quality or outcomes of the specialist care they refer patients for. Similarly, medication management services offered at the CP to recently discharged or ambulatory patients are often done in isolation.^15^ The situation is inconsistent with best TOC practices, encouraging shared knowledge between referring practitioners and patient care continuity during transitions.^16^

Yusuff et al., in their review of care practices for minor ailments among community pharmacists in Nigeria and other developing countries, reported a general lack of definite care protocol and a wide variation in their clerking, counseling, recommendation, and documentation patterns.^17^ We believe that the exclusion of CPs from the national TOC framework and the consequent lack of regulatory oversight may affect the uniformity of referral practices among pharmacists and make quality evaluation difficult. More so, the absence of regulatory provisions may exacerbate the fragile care documentation practice among pharmacists in Nigeria, as most CPs use their care records, where available, for internal auditing and much less for the TOC process.^18^ Taken together, the regulatory gap and its resultant effects continue to impact negatively on the TOC performance of CPs.

Patients' attitude toward referral, primarily referral hesitancy and self‐referral behavior, also hinders the effectiveness of the Nigerian CP's care transition.^14^ Some patients are reluctant to follow up with referrals from CPs. In a feasibility study of a mobile health pharmacy‐based hypertension care in Lagos state, Nigeria, Nelissen et al.^19^ found an overall low uptake of the intervention by patients following mass screening and referral through hospital, pharmacy, and community outreaches. In the study, while 1 in 5 persons screened had elevated blood pressure, only 1 in 34 persons successfully enrolled in the pilot care program.^19^ Notably, however, the authors concluded that pharmacy referrals still recorded higher enrollment rates than referrals from both hospital and community outreach.^19^ Studies have identified possible predisposing and enabling factors for this behavior among patients, including financial challenges, long hospital waiting times, poor understanding of the health system, and poor knowledge about their medical conditions and reason for referral.^13^, ^14^ Notwithstanding, Akodu et al.^20^ reported an overall high satisfaction level with the quality of service received among elderly patients attending nine PHC centers in Lagos state, despite having higher expectations for the services across the five quality domains measured.^17^ Similarly, high satisfaction scores were reported by Auta et al.^21^ among patients consulting pharmacists at CPs in Jos, Nigeria, citing a better understanding of their conditions postvisit.^18^ These findings suggest that, with adequate support, CPs and PHC centers' service delivery can be improved to correct negative perceptions and restore public trust in the PHC system. On the other hand, self‐referral by patients to specialty care for minor ailments that can be effectively managed at CPs or PHC centers causes undue strain on resources at higher levels of care.^6^, ^14^

Furthermore, Nigeria's lack of a centralized health information database also accounts for some problems with referrals at the CP.^15^ The electronic health record system may promote TOC efficiency by facilitating providers' communication, access to vital information, and faster decision‐making. However, the adoption and use of electronic health records in Nigeria is very limited, especially due to infrastructural deficits.^15^ As a result, when patients present at the pharmacy, there are difficulties with accessing all of their necessary medical and medication history, which hampers rapid clinical decisions. This makes the continuation of care, which is the goal of referral, difficult. Also, the new information gathered during the interaction with the pharmacist at the CP is frequently lost when the patient proceeds to another care setting.

Another major barrier is the problem of care provider silos in the Nigerian TOC system, especially in the private sector.^16^ According to Mohammed,^22^ Nigerian health professionals admitted rivalry and interprofessional conflicts are prevalent in the sector, affecting optimal care delivery, and cited disparity in pay, leadership, and lack of collaboration as prominent causes.^16^ Typically, CPs and hospitals work in isolation, and this disconnect extends to the relationship between the practitioners in these two facilities, thereby negatively affecting collaboration and smooth TOC.^16^ Anecdotal evidence suggests that many private hospitals in Nigeria lack significant pharmacist engagement in their operations.^23^ Similarly, some community pharmacists have been implicated in overreaching practices, such as offering prescription‐only medications to patients without a physician's input.^24^ As such, professional silos created by a lack of trust and unhealthy competitive behaviors between care providers may deny patients quality care, where one provider hesitates to refer when due or does so as a mere “handover” devoid of feedback, follow‐up or consideration of patients' needs.

## 3 RECOMMENDATIONS TO REVAMP THE REFERRAL SYSTEM

Addressing the highlighted barriers to effective TOC performance of CPs in Nigeria requires a reform that integrates all factors and players involved in the health referral system of the country. We suggest policy changes focusing on the following aspects:

### 3.1 Restructuring the national care referral framework

A primary recommendation is to expand the national care referral framework to accommodate and institutionalize CPs as member facilities in PHC delivery in Nigeria and strengthen the regulatory guidance on the TOC activities of community pharmacists. Nigerian health policymakers may draw the motivations for this inclusion from the evidence of the impact of CP‐based TOC interventions on patient care outcomes around the world^6^, ^9^ and in Nigeria; for example, the recent successes of the integration of CPs into the differentiated models of care (DMoC) for antiretroviral therapy (ART) for HIV/AIDS in Nigeria. The DMoC strategy was recommended by the WHO in 2015 and involves leveraging private actors, such as CPs in high‐burden countries like Nigeria, to enhance access to ART care, meet increasingly diverse patient needs, and decongest ARV facilities.^25^ According to a retrospective analysis of the DMoC program in four states in Nigeria by Asieba et al.,^26^ the referral of 10,015 stable HIV patients to 244 CPs for ART refill and counseling successfully decongested the 50 participating hospitals, and the initiative was well received by the patients with over 98% retention rate and sustained viral load suppression.^26^

The national guidelines for the reformed TOC framework should define the workflow, stakeholders, incentives, and communication processes expected from the referral system. The reform should also address the factors promoting professional silos in the primary care practice of community pharmacists and bring them into alliance with specialists in higher‐level care facilities. To achieve this, we recommend leveraging digital solutions to catalog health professionals and specialists within each region of the country.^27^ These telehealth systems can accelerate the search and discovery of available specialists within the locale of a given pharmacy premises. Also, such technologies can facilitate targeted referrals and care provider interactions, quicken appointment bookings, bolster patients' follow‐up process, and, ultimately, increase the TOC system's efficiency.^15^, ^27^ It is noteworthy, however, that these digital solutions may face some implementation challenges given the current infrastructural deficits and healthcare practitioners' capacities. Yet, there is optimism that healthcare professionals in Nigeria are generally positively inclined to incorporate digital services into their practice, as evidenced by some studies.^28^ This favorable disposition enhances the feasibility of employing digital solutions for TOC.

### 3.2 Establishment of regional health committees

To improve collaboration among stakeholders, we recommend the establishment of regional health committees among healthcare practitioners to create an avenue for purposeful discourse around strengthening the TOC process within the represented communities. Cooperation and teamwork among health professionals could foster trust, learning opportunities, effective communication, and positive health outcomes for patients.^16^

Furthermore, pharmacist‐to‐pharmacist TOC models operating between the hospital and CPs have been shown to improve patient care outcomes in more robust healthcare systems.^6^, ^7^, ^9^, ^10^ In these models, the medication reconciliation services completed at the hospital on patient discharge are linked with the comprehensive medication management services offered by the CPs as part of the patient's home care.^9^ We recommend adopting this model in Nigeria and expanding it to serve a bilateral pharmacy‐to‐hospital TOC function. This will ensure that the interventions rendered to patients at the CPs get adequately captured in their subsequent hospital‐based care to avoid therapy‐related problems.

### 3.3 Strengthening documentation practices in CPs

Central to a functional TOC system is the appropriate documentation of interventions and efficient transfer of records between practitioners.^7^, ^15^ Community pharmacists in Nigeria should entrench the culture of proper documentation practice and ensure that updated medical records accompany their patients' referral letters during TOC. The guidelines of the proposed model referral system would outline the ideal formatting and details of the referral letter from CPs for uniformity and accuracy.

### 3.4 Healthcare professional education

Healthcare professional training should continue to emphasize and demonstrate the relevance and process of effective TOC. The pharmacy curriculum should be expanded to inculcate tenets of interprofessional education and practice into students early. Patients receiving care at the CPs should be adequately educated about their conditions, made aware of the reason for their referrals, and supported throughout the transition process. This can help allay a patient's fear and mitigate their nonadherence to clinical advice during care transitions. Lastly, we advocate for stronger research efforts on this subject, focused on assessing, designing, and optimizing the TOC activities of CPs in Nigeria.

## 4 CONCLUSION

We address the growing need for the Nigerian healthcare system to incorporate CPs into the TOC model that operates in the country. Given the identified barriers to effective performance, opportunities to enhance the care model abound. Overall, the patient, who is at the core of healthcare, benefits from an efficient, effective, and inclusive TOC system.

## AUTHOR CONTRIBUTIONS

**Adeboye O. Bamgboye**: Conceptualization; writing—original draft. **Ibrahim A. Hassan**: Conceptualization; writing—original draft. **Elijah O. Fatoye**: Conceptualization; writing—review and editing. **Chioma C. Ozuluoha**: Writing—review and editing. **Samson O. Folami**: Writing—review and editing. **Theogene Uwizeyimana**: Supervision; writing—review and editing.

## CONFLICT OF INTEREST STATEMENT

The authors declare no conflict of interest.

## ETHICS STATEMENT

1

All authors agreed to the publication of this manuscript.

## TRANSPARENCY STATEMENT

The lead author Theogene Uwizeyimana affirms that this manuscript is an honest, accurate, and transparent account of the study being reported; that no important aspects of the study have been omitted; and that any discrepancies from the study as planned (and, if relevant, registered) have been explained.

## Data Availability

Not applicable.
